# A Non‐Randomized Comparison of the Addition of New Maudsley Model Workshops for Parents of Adult Women Receiving Multidisciplinary Care for Anorexia Nervosa

**DOI:** 10.1002/eat.24577

**Published:** 2025-10-15

**Authors:** Cristiano Dani, Eleonora Rossi, Emanuele Cassioli, Valentina Zofia Cordasco, Alice Roscioli, Giulia Selvi, Livio Tarchi, Luca Zompa, Sandra Moretti, Maria Rita Troiani, Stefano Lucarelli, Valentina Cardi, Valdo Ricca, Giovanni Castellini

**Affiliations:** ^1^ Department of Health Sciences University of Florence Florence Italy; ^2^ UFS Eating Disorders ASL Toscana Centro Florence Italy; ^3^ Department of General Psychology University of Padova Padua Italy; ^4^ Centre for Research in Eating and Weight Disorders Institute of Psychiatry, Psychology and Neuroscience, King's College London UK

**Keywords:** anorexia nervosa, carers, New Maudsley Model, outcome, parents

## Abstract

**Objective:**

The impact of the add‐on of New Maudsley Model (NMM) training workshops for carers of individuals with eating disorders (EDs) on clinical outcomes in adults with anorexia nervosa (AN) undergoing enhanced cognitive behavior therapy (CBT‐E) remains unexplored. The outcomes of females with AN aged 18 and 35 years, treated with CBT‐E, were compared between those whose parents agreed to attend NMM workshops and those whose parents declined participation.

**Method:**

Psychopathology was assessed up to 1‐year follow‐up. Baseline differences were analyzed using analysis of covariance (ANCOVA), while longitudinal changes were evaluated with generalized additive mixed models (GAMMs).

**Results:**

At admission, individuals in the NMM group had higher levels of ED psychopathology, body uneasiness, and alexithymia. At 1‐year follow‐up, both groups improved significantly, and between‐group comparisons revealed significant effect sizes in favor of the NMM group.

**Discussion:**

Parental participation in NMM workshops was associated with more favorable treatment outcomes in adults with AN undergoing CBT‐E. These findings suggest the potential relevance of integrating family participation into adult AN treatment.

**Summary**

- Adult individuals with anorexia nervosa (AN) whose parents participated in New Maudsley Model (NMM) workshops exhibited greater baseline severity in eating disorder (ED) psychopathology, body uneasiness, and alexithymia compared to those whose parents declined to participate.
- The integration between NMM workshops and enhanced cognitive behavior therapy (CBT‐E) led to greater improvements in body mass index (BMI), ED psychopathology, general psychopathology, body uneasiness, emotional dysregulation, and alexithymia at 1‐year follow‐up in adult individuals with AN compared to individuals whose parents did not receive any support.
- These findings highlight the potential relevance of parental involvement in the treatment of adult individuals with AN.

## 1 Introduction

Anorexia nervosa (AN) is a severe psychiatric disorder with a broad impact on mental and physical health (Larsen et al. 2024). The high prevalence of psychiatric and organic comorbidities (Solmi et al. 2024), the chronic nature of the disease (Keski‐Rahkonen and Mustelin 2016), and the challenges in finding proper personalized treatment (Schmidt et al. 2025) contribute to the increased risk of hospitalization among individuals with AN (Castellini et al. 2023). Moreover, eating disorders (EDs), including AN, typically emerge during childhood or early adulthood, with most cases developing before the age of 25 (Solmi et al. 2022). A prolonged duration of untreated illness could further exacerbate these risks, leading to worse clinical outcomes and making recovery more challenging (Gumz et al. 2023). Conversely, early weight gain was identified as a crucial predictor of long‐term recovery (Maurel et al. 2024).

Enhanced cognitive behavioral therapy (CBT‐E) is the most widely recommended treatment for adults with AN (Fairburn 2008). It is a disorder‐specific form of CBT, applicable across the full range of ED diagnoses, and has demonstrated effectiveness for improving weight restoration and reducing psychopathology (Atwood and Friedman 2020; Dalle Grave et al. 2020; De Jong et al. 2020). Predictors of positive outcomes following intensive CBT‐E include early weight gain and lower baseline ED psychopathology, while longer illness duration and higher clinical severity predict poorer results (Calugi et al. 2025). Despite its robust evidence base, CBT‐E currently offers limited guidance on the involvement of caregivers, even though family participation is increasingly recognized as potentially beneficial in the treatment of adults with AN (Treasure et al. 2021).

Caregivers play a crucial role as they are often the first ones to detect ED symptoms (Wilksch 2023), and their involvement is also important in supporting the recovery of individuals with EDs. The importance of the family environment in recovery has been widely recognized, with family functioning—particularly communication and problem‐solving—identified as key factors in ED psychopathology (Monteleone et al. 2024). However, caregivers of individuals with AN may often exhibit accommodating and enabling behaviors (Pedersen et al. 2024), potentially reinforcing disordered eating patterns. These behaviors may represent maladaptive coping strategies used to manage the distress and complexity of the disease, further emphasizing the need for structured support interventions (Rhind et al. 2016; Stefanini et al. 2019; Zeiler et al. 2025).

Psychoeducation has been integrated into treatment for EDs as a proven strategy to reduce caregiver burden and improve clinical outcomes for children and adolescents (Kurnik Mesarič et al. 2024). Moreover, early parenting group interventions have demonstrated positive effects on body mass index (BMI) and ED psychopathology at 6 months (Rosello et al. 2021). Skills‐based workshops and psychoeducational interventions have also been shown to reduce ED symptoms and psychological distress in adults (Sepúlveda et al. 2019), and caregiver skills training has improved ED psychopathology and quality of life at 6 months post‐discharge even in adult individuals with severe AN (Hibbs et al. 2015). Nevertheless, caregiver‐based interventions remain primarily designed for children and adolescents with AN (Cripps et al. 2024; Fernández García and Quiles Marcos 2024). In this regard, a recent study highlighted the lack of sufficient caregiver support in adult ED services, emphasizing the need for smoother transitions from child and adolescent to adult care to enhance both patient and caregiver outcomes (Cribben et al. 2025). This highlights a significant gap in research and intervention strategies tailored to caregivers of adults with EDs, despite the demonstrated benefits of such approaches in younger populations. Therefore, the current body of literature is not sufficient to draw conclusions about their effectiveness in adult individuals.

Despite this gap, the New Maudsley Model (NMM), an evolution of traditional family‐based treatment (FBT), offers a distinct approach by incorporating caregivers into the support process for adult individuals with EDs, providing families with training workshops to enhance communication, deliver emotional support, and offer effective assistance without enabling disordered behaviors (Treasure et al. 2015). The positive effect of NMM training workshops on communication and relationships with carers was confirmed by the findings of a qualitative study on adult individuals with AN, which also underlined the importance of the support by carers in their motivation toward recovery (Goddard et al. 2011). However, further research is needed to clarify their impact on clinical outcomes when integrated into evidence‐based individual treatments such as CBT‐E.

Expanding on this, the present study aimed to investigate the role of caregiver support in the treatment of adult individuals with AN. The NMM was selected for its structured and time‐limited format (Treasure et al. 2015), consisting of seven standardized and structured sessions promoting autonomy and reducing enabling behaviors, which facilitate integration into multidisciplinary treatment plans within public healthcare services. Specifically, the study assessed whether participation in NMM training workshops for caregivers was associated with a different outcome in individuals treated with CBT‐E at 1‐year follow‐up. To eliminate potential confounding effects, the possible presence of psychopathological differences at baseline between those whose parents accepted or declined participation in the NMM workshops was considered. By addressing family dynamics that are not specifically targeted by CBT‐E (Dalle Grave et al. 2019), the NMM may help remove family‐level barriers that can otherwise limit the effectiveness of the individual treatment. In this context, and considering their basis in the CBT‐E framework as well as their potential to facilitate treatment, and thus the achievement of core therapeutic targets, it was hypothesized that participation in the NMM workshops would be associated with greater improvements in the main clinical outcomes over 1 year, including BMI and ED‐related psychopathology.

Given the challenges of treating individuals with AN (Schmidt et al. 2025), integrating structured caregiver support may represent a relevant addition to standard individual treatments such as CBT‐E.

## 2 Method

### 2.1 Study Design

The study participants were selected from individuals diagnosed with AN who were referred to the Eating Disorder Unit of the Psychiatry Unit of Careggi University Hospital (Florence, Italy) between 2021 and 2024. Enrollment required informed consent from both the individuals with AN and their parents, ensuring mutual agreement to participation. The inclusion criteria were the following: female gender, age between 18 and 35 years, a current diagnosis of AN at the time of recruitment according to the Diagnostic and Statistical Manual of Mental Disorders, Fifth Edition (DSM‐5; American Psychiatric Association 2013), cohabiting with parents who were enrolled in the current study at admission, and the provision of written informed consent. The exclusion criteria were: ending cohabitation during the study period, illiteracy, intellectual disability, or other conditions that could hinder the proper understanding of the study protocol and completion of the questionnaires. Only parents were included as caregivers in this study to ensure consistency in the type of familial support examined. Parental cohabitation was required to capture ongoing, day‐to‐day caregiving dynamics, which may differ significantly from those of non‐cohabiting caregivers.

A total of 253 individuals with AN were initially referred for participation in the study. Of these, 18 declined to participate, 49 did not meet the inclusion criteria, and 30 individuals (16 lost to follow‐up and 14 dropouts) did not complete any evaluation beyond the initial assessment. The final sample included 186 individuals, who were selected for the analyses. A CONSORT flow diagram (Hopewell et al. 2025) is presented in Figure S1, illustrating the recruitment process.

### 2.2 Study Population

The study population consisted of two groups: one group included individuals whose parents consented to participate in the training workshops (NMM; *n* = 65), and the other group included individuals whose parents declined participation (no NMM; *n* = 121). The entire sample (100%) consisted of individuals of White ethnicity. Both groups received individual CBT‐E treatment, with the experimental group additionally participating in NMM training workshops for caregivers. The two groups were compared on clinical characteristics and psychopathology at baseline (T0), 6 months (T6), and 1 year after baseline (T12). The longitudinal time points may have varied (±1 month) due to factors such as organizational constraints within the unit and the availability of individuals. The study protocol was reviewed and approved by the Institutional Ethics Committee (Comitato Etico Regione Toscana—Area Vasta Centro).

### 2.3 Data Collection

All participants underwent a clinical interview to verify their eligibility for the study. General characteristics and anthropometric data were collected through a brief clinical interview conducted during the initial outpatient evaluation, prior to any therapeutic intervention. Data were collected by psychiatrists with expertise in diagnosing and treating EDs at each timepoint (T0, T6, T12).

The following questionnaires were administered to comprehensively assess key clinical dimensions relevant to the psychopathology of AN:

- Eating Disorder Examination Questionnaire version 6.0 (EDE‐Q; Calugi et al. 2015; Fairburn and Beglin 1994), a questionnaire assessing the behavioral and cognitive features of EDs divided into four subscales: restraint, eating concern, weight concern, and shape concern. In the recruited population, internal consistency was excellent for the global score (Cronbach's *α* = 0.97), with high reliability also observed across the subscales: restraint (*α* = 0.88), eating concern (*α* = 0.86), weight concern (*α* = 0.91), and shape concern (*α* = 0.94).
- The Body Uneasiness Test, part A (BUT‐A; Cuzzolaro et al. 2006). It provides a Global Severity Index (BUT‐A GSI), that is the average of the individuals scores reported for each of the items (range 0–5), and five subscales: Weight phobia, body image concerns, avoidance, compulsive self‐monitoring, and depersonalization. Higher scores indicate greater body uneasiness. This measure demonstrated excellent internal consistency in this study for the GSI (included in the analyses), with a Cronbach's *α* = 0.92.
- The short form of the Childhood Trauma Questionnaire (CTQ; Sacchi et al. 2018), a 28‐item questionnaire including five subscales, which evaluate five specific forms of childhood trauma (i.e., sexual abuse, physical abuse, physical neglect, emotional abuse, and emotional neglect), and a total score. The Cronbach's alpha computed for the total score (employed in the analyses) in the current sample was good (*α* = 0.74).
- The Difficulties in Emotion Regulation Scale (DERS; Gratz and Roemer 2004) evaluates trait‐level emotion dysregulation through 36 items on a five‐point scale. Six subscales (non‐acceptance of emotional responses, difficulties engaging in goal‐directed behavior, limited access to emotion regulation strategies, impulse control difficulties, lack of emotional clarity, and lack of emotional awareness) can be obtained from the sum of the appropriate items, while the total score is obtained by summing all items. In the current sample, internal consistency for the total score (utilized in the analyses) was excellent (Cronbach's *α* = 0.90).
- The Symptom Checklist‐90‐Revised (SCL‐90‐R; Prunas et al. 2012). This instrument encompasses nine primary dimensions of symptomatology (somatization, obsessive‐compulsive, interpersonal sensitivity, depression, anxiety, hostility, phobic anxiety, paranoid ideation, and psychoticism), along with three global distress indexes (Global Severity Index—GSI), positive symptom distress index (PSDI), and positive symptom total (PST), serving as indicators of emotional disorder. The Cronbach's alpha calculated in the recruited population for the GSI (used in the analyses) was satisfactory (*α* = 0.79).
- The short version of the Toronto Alexithymia Scale (TAS‐20; Bressi et al. 1996) measures alexithymia through 20 items. It provides three sub‐dimensions (i.e., difficulties in identifying emotions, difficulties in describing emotions, and externally oriented thinking), and a total score (used in the analyses). Cronbach's alpha for the total score was 0.88.
- The Attachment Style Questionnaire (ASQ; Fossati et al. 2003) is a self‐report measure assessing adult attachment styles. It consists of five subscales: confidence in relationships, discomfort with closeness, need for approval, preoccupation with relationships, and relationships as secondary. In the recruited population, the questionnaire demonstrated acceptable internal consistency: confidence (*α* = 0.75), discomfort with closeness (*α* = 0.72), need for approval (*α* = 0.73), preoccupation with relationships (*α* = 0.74), and relationships as secondary (*α* = 0.70).

### 2.4 Intervention

All individuals received treatment within a multidisciplinary setting, which included regular psychiatric and dietetic evaluations as well as internal medicine consultations when needed. In addition, all individuals underwent an initial admission to an outpatient program to facilitate engagement with the services and ensure access to routine diagnostic assessments, as outlined in the regional care pathway for the Florence area (Castellini et al. 2023). This standard care, referred to here as Treatment as Usual (TAU), consisted of a coordinated program in which all participants received the same baseline therapeutic framework; only those whose parents agreed to participate in the NMM program received additional caregiver support. All individuals underwent at least 40 individual sessions of CBT‐E (Fairburn 2008), initially scheduled once or twice per week during the intensive phase. In the final treatment phase, sessions were spaced out to every 2 or 3 weeks based on individual needs. All CBT‐E sessions were delivered by certified CBT‐E trainers, and weekly supervision meetings were held to ensure treatment fidelity and consistency across therapists. The median number of CBT‐E sessions per patient was 42 (range 39–52). In addition to psychotherapy, patients received individualized dietary counseling from specialist dietitians, focused on restoring nutritional balance, supporting healthy eating patterns, and preventing relapses. Psychiatric monitoring was provided regularly to evaluate mental health status and adjust treatment plans. Individuals meeting DSM‐5 criteria for major depressive disorder (American Psychiatric Association 2013) were prescribed selective serotonin reuptake inhibitors (SSRIs) based on clinical judgment and in accordance with National Institute for Health and Care Excellence guidelines (NICE 2022). Medical assessments included regular weight checks, vital signs monitoring, and laboratory tests to ensure medical stability. All interventions were part of TAU and were not affected by study participation.

All the parents of individuals included in the study were proposed at the time of admission to take part in NMM training workshops, which aimed to equip them with practical tools and strategies to better support their family members with an ED (Treasure et al. 2015). This program consisted of seven structured sessions, each lasting 2 h, and was conducted by team members with expertise in the fields of EDs. Parents started the NMM workshops concurrently with the initial phase of CBT‐E treatment, ensuring alignment between caregiver support and the patient's therapeutic process from the outset. Facilitators of the training workshops recommended that participants purchase the written NMM manual in Italian (Stefanini et al. 2024).

### 2.5 Statistical Analyses

Continuous variables were reported as mean and standard deviation. All tests are reported with effect sizes and 95% confidence intervals (CIs) where appropriate. Comparisons were conducted among individuals with AN to assess potential differences in psychopathology severity or disease status at admission, based on their parents' decision to participate in NMM training workshops, to contextually start a structured family‐based intervention. Participants were divided into two groups (NMM; no NMM) based on whether their parents agreed to attend the NMM training workshops. Comparisons were performed on general characteristics and psychometric data using analysis of covariance (ANCOVA), adjusting for age and BMI to account for potential baseline differences. Categorical variables were compared across groups using Pearson's Chi‐squared test with Yates' continuity correction.

Based on the hypothesis that the decision of parents to participate or not in the NMM training workshops could create two distinct subgroups of individuals with AN, a longitudinal analysis was conducted under an intention‐to‐treat (ITT) framework (Gupta 2011) to assess future outcomes at a 1‐year follow‐up. The aim was to evaluate whether NMM training workshops could exert improvements in BMI and psychopathology over time. The method of choice for this longitudinal analysis was Generalized additive mixed models (GAMMs), a flexible statistical approach that extends linear mixed models by allowing for nonlinear relationships between predictors and outcomes, accounting for individual variability over time by incorporating random effects while enabling the modeling of complex trajectories (Wood 2017). This approach was particularly suited for the study, as it allowed for the evaluation of both linear and nonlinear effects of parental participation on BMI and psychometric measures, with the application of a random intercept. GAMMs were selected over GLMMs or traditional LMMs because they allow for greater flexibility in modeling complex, potentially nonlinear trajectories without imposing restrictive assumptions on the shape of change over time. While GLMMs can accommodate some nonlinear effects, they generally require the shape of the curve to be specified a priori (e.g., polynomial or logarithmic terms), which increases the risk of model misspecification. By contrast, GAMMs estimate the smooth function of time directly from the data, providing a more robust and less assumption‐driven approach, consistent with the design of our study (Wood 2017). This was particularly important in the present cohort, where treatment progress did not necessarily follow a linear or predefined pattern. In all GAMMs, time was entered as a smooth term based on thin plate regression splines, which are considered optimal; a modified smoothing penalty was used in order to allow the smooth term to be shrunk to 0 and avoid overfitting (Wood 2017). Moreover, group membership (participation in the NMM training workshop) was entered as a fixed effect, and interaction with time was modeled explicitly by computing separate smooth functions for each group. This allowed the model to flexibly capture and compare group‐specific trajectories over time, thus effectively representing a Group × Time interaction. Both follow‐up time points (T6 and T12) were incorporated into the model simultaneously. Unlike traditional linear mixed models that provide separate effect estimates for each time point, the GAMM framework provides a single, overall estimate of the temporal effect by modeling time as a nonlinear smooth function. This approach thus captures the entire longitudinal trajectory without focusing on discrete time points. In order to compare the two groups on changes over time, adjusted changes (T12 − T0, using model‐based estimates) were calculated for each variable under investigation, and the two groups were compared with one another by computing a between‐group effect size (Δ Cohen's *d*) and its corresponding confidence interval.

An a priori power analysis was also conducted using repeated‐measures ANOVA assumptions (two groups, three time points, correlation among repeated measures of 0.4, nonsphericity correction *ε* = 1). Assuming a small‐to‐moderate effect size (*f* = 0.20), an alpha level of 0.05, and a desired power of 0.95, the analysis indicated that a total sample size of 80 participants would be required to reliably detect a Group × Time interaction, thus guiding recruitment targets for the study (Faul et al. 2007).

As significant differences at baseline between groups were expected, the baseline score for each psychometric score, as well as the baseline values of age and BMI, were included as covariates to ensure the reliability of the longitudinal data. This approach was employed to control for initial group differences and to enhance the reliability and interpretability of the longitudinal outcomes by isolating the effect of caregiver intervention from pre‐existing disparities. In accordance with common guidelines for statistical reporting, the *F* value and its corresponding *p* value, as well as effective degrees of freedom (EDF), have been provided for all smooth terms. In this context, EDF can be used as a proxy for measuring the nonlinearity of the relationship between the variables in the model: a value less than or equal to 1 indicates a substantial linear trend of the dependent variable over time, while a value greater than 1 indicates an increasingly curved longitudinal trend. For significant time smooth terms, the first derivatives of the fitted trend and their respective 95% CI were computed using standard theory: a time interval where the CI on the first derivative did not include 0 was considered to be a period of statistically significant change (Simpson 2018). In addition, control analyses were conducted to assess the role of potentially influential covariates, specifically duration of illness, SSRI medication use, and number of CBT‐E sessions. Given the lack of a unified definition of recovery (Bardone‐Cone et al. 2018), two definitions were used, aligning with the criteria for AN as per DSM‐5 (American Psychiatric Association 2013): partial remission, defined as achieving a BMI ≥ 18.5; and full remission, defined as achieving both a BMI ≥ 18.5 and an EDE‐Q global score < 2.5, sustained for at least 3 months. For each patient, remission status was determined based on the presence or absence of these criteria within the follow‐up window, ensuring that remission classification was consistent at the individual level. Logistic regression analyses were conducted to assess whether group membership (participation vs. non‐participation in NMM workshops) predicted full or partial remission, adjusting for baseline BMI and EDE‐Q global score.

The statistical analysis was performed with R statistical software v4.5.1, with the following libraries: nlme (Pinheiro et al. 2021), mgcv (Wood 2011).

## 3 Results

### 3.1 Group Characteristics at Admission

The results revealed significant differences at the time of admission between individuals with AN whose parents participated in NMM training workshops (NMM group) and those whose parents declined to participate (no NMM group).

No significant differences emerged in terms of age and BMI between groups, while individuals with parental participation exhibited greater severity across several psychopathological measures. Specifically, they reported significantly higher levels of ED psychopathology (EDE‐Q), including dietary restraint, eating concern, shape concern, and global score. Additionally, they showed higher levels of body uneasiness (BUT‐A GSI) and alexithymia (TAS‐20 total score). Regarding attachment style (ASQ), individuals in the NMM group showed a lower confidence in relationships and an increased preoccupation with relationships. Conversely, no significant differences emerged in childhood maltreatment (CTQ), general psychopathology (SCL‐90‐R), or emotional dysregulation (DERS). The results are reported in Table 1.

**TABLE 1:** Sample descriptives and psychopathological correlates at admission time across adult individuals with AN whose parents have (NMM) or have refused (no NMM) to participate in NMM training workshops.

	NMM	No NMM	df	*F*	*η* ^2^	95% CI
Age	20.34 ± 3.35	20.26 ± 3.13	184	0.03	0.00	[0.00, 0.05]
BMI (kg/m^2^)	15.98 ± 1.47	16.11 ± 1.47	184	0.27	0.00	[0.00, 0.04]
Duration of illness	2.83 ± 2.37	2.93 ± 3.14	184	0.05	0.00	[0.00, 0.02]
EDE‐Q dietary restraint	4.33 ± 1.56	3.38 ± 1.89	182	10.37**	0.06	[0.01, 0.14]
EDE‐Q eating concern	3.92 ± 1.21	3.05 ± 1.60	182	12.79***	0.07	[0.01, 0.16]
EDE‐Q weight concern	4.16 ± 2.72	3.60 ± 2.25	182	2.30	0.01	[0.00, 0.07]
EDE‐Q shape concern	4.39 ± 1.43	3.68 ± 1.76	182	7.59**	0.04	[0.01, 0.12]
EDE‐Q global score	4.20 ± 1.56	3.43 ± 1.56	182	10.07**	0.06	[0.01, 0.14]
BUT‐A GSI	3.08 ± 0.83	2.57 ± 1.16	182	7.24	0.05	[0.01, 0.15]
CTQ total score	41.46 ± 14.58	43.25 ± 14.97	182	0.45	0.00	[0.00, 0.05]
DERS total score	113.45 ± 28.33	107.47 ± 28.07	182	1.53	0.01	[0.00, 0.08]
SCL‐90‐R GSI	1.78 ± 0.67	1.55 ± 0.75	182	3.81	0.02	[0.00, 0.09]
TAS‐20 total score	63.43 ± 11.4	55.64 ± 13.35	182	8.98**	0.09	[0.01, 0.21]
ASQ C	24.19 ± 7.83	27.65 ± 7.34	182	6.93**	0.05	[0.01, 0.14]
ASQ DC	39.58 ± 11.08	37.23 ± 9.55	182	1.88	0.01	[0.00, 0.08]
ASQ RS	21.15 ± 7.94	19.09 ± 8.26	182	1.96	0.01	[0.00, 0.08]
ASQ NA	30.60 ± 7.40	28.38 ± 7.90	182	2.93	0.02	[0.00, 0.09]
ASQ PR	34.31 ± 6.86	30.65 ± 8.21	182	8.40**	0.06	[0.01, 0.15]

*Note*: Age‐ and BMI‐adjusted ANCOVAs are presented. *F* values are presented with corresponding significance levels and effect sizes (*η* ^2^) along with 95% confidence intervals (**p < 0.01, ***p < 0.001).

Abbreviations: ASQ, Attachment Style Questionnaire; BMI, body mass index; BUT‐A, Body Uneasiness Test‐A; C, confidence; CTQ, Child Trauma Questionnaire; DC, discomfort with closeness; DERS, Difficulties in Emotion Regulation Scale; df, degrees of freedom; EDE‐Q, Eating Disorder Examination Questionnaire; GSI, Global Severity Index; NA, need for approval; PR, preoccupation with relationships; RS, relationships as secondary; SCL‐90, Symptom Checklist‐90‐Revised; TAS‐20, Toronto Alexithymia Scale—20.

### 3.2 Longitudinal Impact of NMM Training Workshops on Clinical Outcomes

The GAMM models examined the longitudinal trajectories of multiple clinical outcomes (BMI, EDE‐Q, DERS, SCL‐90‐R, TAS‐20) at 1‐year follow‐up after admission, considering the effect of group membership based on parental acceptance or refusal to participate in NMM training workshops (NMM vs. no NMM groups).

The results indicated a significant increase in BMI over time in both groups, with a greater effect size of the restoration BMI in the NMM group compared to the no NMM group. Similarly, individuals in the NMM group showed a greater effect size in terms of reduction over time in ED psychopathology (EDE‐Q) compared to the no NMM group and similarly showed linear greater improvements in general psychopathology (SCL‐90‐R), body uneasiness (BUT‐A), and emotional dysregulation (DERS). Significant improvements in alexithymia (TAS‐20) were observed only in the NMM group.

Importantly, between‐group comparisons at T12 revealed significant effect sizes in favor of the NMM group across all outcomes, suggesting a potential advantage of NMM training workshops for clinical improvements.

Table 2 reports the observed means ± SD for each group at T0, T6, and T12, as well as model‐derived within‐group effect sizes (T0–T12) and between‐group effect sizes at T12 (Cohen's *d* with 95% CI). These results are graphically illustrated in Figures S2 and S3. The longitudinal Group × Time estimates derived from the GAMM models are presented in Table 3. All analyses were adjusted for baseline scores of each clinical outcome, as well as for age and BMI.

**TABLE 2:** Changes in clinical outcomes across groups (NMM, no NMM) at baseline (T0), 6 months (T6), and 12 months (T12).

Group × Time	Group	T0	T6	T12	Within‐group *d* (95% CI)	Between‐group Δ *d* (95% CI)
BMI (kg/m^2^)	NMM	15.98 ± 1.47	17.11 ± 1.46	17.27 ± 1.92	1.10 [0.86, 2.34]	0.16 [0.14, 0.17]
	No NMM	16.11 ± 1.47	17.55 ± 1.74	17.67 ± 1.52	0.93 [0.77, 2.24]	
EDE‐Q global score	NMM	4.20 ± 1.56	3.57 ± 1.50	2.89 ± 2.01	0.81 [1.01, 1.91]	−0.33 [−0.36, −0.31]
	No NMM	3.43 ± 1.56	2.75 ± 1.65	2.07 ± 1.76	0.44 [0.62, 1.51]	
BUT‐A GSI	NMM	3.08 ± 0.83	2.45 ± 1.30	1.87 ± 1.80	0.58 [0.34, 0.89]	−0.05 [−0.06, −0.04]
	No NMM	2.57 ± 1.16	2.18 ± 1.18	1.62 ± 1.25	0.34 [0.29, 0.80]	
DERS total score	NMM	113.45 ± 28.33	99.80 ± 40.67	88.20 ± 30.51	0.25 [0.16, 0.33]	−0.02 [−0.03, −0.02]
	No NMM	107.47 ± 28.07	94.50 ± 28.99	85.10 ± 27.83	0.15 [0.07, 0.24]	
SCL‐90‐R GSI	NMM	1.78 ± 0.67	1.58 ± 0.68	1.37 ± 0.68	0.95 [0.24, 0.62]	−0.02 [−0.03, −0.02]
	No NMM	1.55 ± 0.75	1.39 ± 0.71	1.24 ± 0.68	0.36 [0.12, 0.44]	
TAS‐20 total score	NMM	63.43 ± 11.4	59.5 ± 11.0	53.8 ± 10.9	0.67 [0.19, 1.29]	−0.61 [−0.68, −0.55]
	No NMM	55.64 ± 13.35	54.5 ± 13.1	53.1 ± 13.3	0.62 [−0.94, 1.08]^a^	

*Note*: Values are means ± standard deviations at each time point. Effect sizes are reported as Cohen's *d* with 95% confidence intervals, corrected for correlated observations. Within‐group effect sizes refer to changes from T0 to T12 and are calculated based on model‐estimated means and standard deviations (i.e., adjusted for covariates). All within‐group changes were significant except for TAS‐20 in the no NMM group (^a^non‐significant). Between‐group effect sizes (Δ Cohen's *d*) represent the difference between groups in the variation over time (T12 − T0), based on model‐estimated means and standard deviations (i.e., adjusted for covariates). All between‐group changes were statistically significant.

Abbreviations: BMI, body mass index; BUT‐A, Body Uneasiness Test‐A; DERS, Difficulties in Emotion Regulation Scale; EDE‐Q, Eating Disorder Examination Questionnaire; GSI, Global Severity Index; SCL‐90‐R, Symptom Checklist‐90‐Revised; TAS‐20, Toronto Alexithymia Scale—20.

**TABLE 3:** Generalized additive mixed models (GAMMs) estimates for between‐group differences over time (NMM vs. no NMM), adjusted for age, BMI, and baseline value.

	Group fixed effect	NMM		No NMM	
		*F*	EDF	*F*	EDF
BMI (kg/m^2^)	−0.14, [−0.64, 0.36]	2.15***	0.96	0.99***	0.99
EDE‐Q global score	0.01, [−0.21, 0.23]	3.77***	1.06	2.56***	1.03
BUT‐A GSI	0.01, [−0.13, 0.15]	0.98***	0.96	1.67***	0.93
DERS total score	−0.55, [−4.94, 3.83]	1.22***	0.98	1.02***	0.97
SCL‐90‐R GSI	0.04, [−0.06, 0.13]	0.99***	0.97	1.52***	1.00
TAS‐20 total score	2.20, [−2.10, 6.50]	0.63***	0.87	0.24	0.69

*Note*: Values are GAMM estimates for the fixed effect of group (NMM vs. no NMM) on each outcome, reported as *β* with 95% confidence intervals (***p < 0.001), adjusted for baseline values, age, and BMI. *F* values are derived from the GAMMs. Effective degrees of freedom (EDF) reflect the flexibility of the nonlinear time smooth for each group: EDF ≤ 1 indicates a predominantly linear trajectory, while EDF > 1 indicates increasing nonlinearity.

Abbreviations: BMI, body mass index; BUT‐A, Body Uneasiness Test‐A; DERS, Difficulties in Emotion Regulation Scale; EDE‐Q, Eating Disorder Examination Questionnaire; GSI, Global Severity Index; SCL‐90‐R, Symptom Checklist‐90‐Revised; TAS‐20, Toronto Alexithymia Scale—20.

### 3.3 Secondary and Control Analyses

Logistic regression analysis for partial remission revealed that the NMM group was significantly associated with greater odds of remission (OR = 3.53, 95% CI [1.04, 11.90]), after controlling for baseline BMI and EDE‐Q scores. In contrast, the analysis for full remission showed that there were no group differences in full remission status (OR = 1.43, 95% CI [0.49, 4.15]). No significant differences in SSRI medication use were found across groups (*χ* ^2^ = 0.56, *φ* = 0.06; 95% CI [0.00, 0.21]). Furthermore, duration of illness, SSRI use, and number of CBT‐E sessions did not significantly affect any clinical outcomes. The results of secondary analyses are available in Tables S1–S3.

## 4 Discussion

This study provides preliminary evidence that adding NMM training workshops for parents may be associated with additional clinical benefits for individuals with AN under CBT‐E. While CBT‐E is effective in targeting the core psychopathology of AN (Atwood and Friedman 2020), it offers limited guidance on structured caregiver involvement. Thus, the NMM training workshops might serve as a complementary intervention, aiming to optimize the recovery environment and extend the benefits of CBT‐E.

In our study, we also found the presence of pre‐existing differences between individuals whose parents agreed or not to participate in the workshops and emphasized that, even accounting for these differences, participation in the workshops provided benefits.

Specifically, at admission, individuals whose parents accepted to participate in NMM training workshops exhibited higher severity of ED psychopathology, body uneasiness, and alexithymia. These results suggest that caregiver engagement in psychoeducational programs may be sought in response to greater disease severity, which might reflect higher levels of concern and need for intervention. Prior research has shown that perceptions of illness severity, control, and responsibility can strongly influence parental distress and involvement in the recovery process (Marchetti and Sawrikar 2024). Furthermore, the level of support caregivers provide in the recovery process of their child may also be influenced by their subjective experiences and beliefs about AN (Matthews et al. 2018) as well as the burden of caregiving, with guilt and self‐shame acting as key drivers (Matthews et al. 2018; Treasure et al. 2001).

In addition, individuals whose parents participated in NMM workshops showed more maladaptive attachment profiles, characterized by lower confidence in relationships and greater preoccupation with relational aspects. These findings may reflect ambivalent attachment, characterized by exaggerated and more intense expressions of negative affect (Mikulincer and Shaver 2007). This may result in a higher caregiver burden, consequently leading to the perception of a greater need to participate in the NMM workshops.

The longitudinal analyses suggested a potential beneficial association between participation in NMM training workshops and clinical outcomes in adults with AN. Indeed, over a 1‐year follow‐up, individuals whose parents participated in NMM training workshops exhibited significant between‐group comparisons effect sizes, reflecting greater improvements in BMI and ED psychopathology compared with patients whose parents did not participate. Similar effects were also observed for general psychopathology, body uneasiness, emotional dysregulation, and alexithymia. These results were adjusted for each psychometric variable at baseline, as well as for age and BMI, ensuring that the observed effects in the treatment group were not due to the higher severity at baseline but specifically to the intervention. Importantly, these results were observed in the context of treatment with CBT‐E, suggesting that the NMM training workshops may complement and extend its effects by creating a more supportive family environment (Treasure et al. 2015) and addressing dynamics that individual therapy may not directly cover (Dalle Grave et al. 2019), thereby creating conditions more favorable for recovery.

This interpretation is consistent with prior research, indicating that structured caregiver support enhances recovery by reducing the emotional burden and improving disease management strategies (Baudinet et al. 2021). Specifically referring to NMM, these results support the findings of a previous qualitative study, confirming the importance of caregiver support in motivating individuals with EDs toward recovery (Goddard et al. 2011). Notably, patients whose parents participated in NMM were more likely to achieve partial remission, indicating a possible role of caregiver engagement in facilitating weight restoration. However, the lack of difference in full remission rates may reflect the more stringent criteria for full remission and the relatively limited duration of follow‐up. Notably, the observed improvements in clinical outcomes are consistent with those reported in previous studies using similar methodologies in naturalistic, real‐world settings, further supporting the external validity of our findings (Cassioli et al. 2022; Kessler et al. 2022; Rossi et al. 2022; Van Den Berg et al. 2022).

These results highlighted the importance of integrating family interventions into the treatment of adult individuals with AN, addressing a significant gap in the literature. While the involvement of caregivers in the recovery process has been extensively studied in child and adolescent populations, its applicability to adults has received considerably less attention (Cripps et al. 2024). Furthermore, numerous challenges must be addressed to ensure a seamless transition from child and adolescent services to adult care settings (Herpertz‐Dahlmann et al. 2021). Despite the availability of effective treatments such as CBT‐E, AN remains highly resistant to recovery, with relapse rates and chronicity remaining elevated (Solmi et al. 2024). Many individuals continue to experience persistent symptoms despite these interventions, underscoring the need for additional, integrated support systems that target both the psychological and relational aspects of the disorder (Davey et al. 2023; Treasure and Livanou 2024). The integration of family interventions may also be particularly relevant for individuals at different stages of illness chronicity, including those with longstanding illness who often express the need for more personalized and flexible approaches, as suggested in recent stepped‐care frameworks (Byrne and Fursland 2024). This study contributes to this area by suggesting that parental participation in NMM training workshops may be associated with improved patient outcomes in the context of CBT‐E for adult individuals with AN, reinforcing the need for structured integration of caregivers into tailored treatment programs for EDs (Treasure et al. 2021).

However, some limitations must be acknowledged. First, the study focused exclusively on female participants of White ethnicity, which limits the generalizability of the findings to more diverse populations. Second, although key covariates (age, BMI, baseline severity) were controlled, other potential confounders such as socioeconomic status were not assessed. Third, group assignment was non‐random, and participation in the caregiver intervention was based on parental willingness, potentially introducing selection bias. Baseline differences in psychopathology and other clinical variables were observed between groups. To address this, baseline psychometric scores, along with age and BMI, were included as covariates in the analyses to reduce the impact of these initial disparities on the outcome measures. In addition, no data were collected on caregiver demographics (e.g., education, income), limiting insight into factors influencing participation. A further limitation is the lack of demographic and clinical information for patients who declined participation, which prevents assessment of the representativeness of the sample and may introduce selection bias. Moreover, the study was not pre‐registered, which should be considered when interpreting the findings. Finally, the reliance on self‐report measures, despite their widespread use in psychiatric research, may have introduced response biases. Future research should aim to replicate these findings in more diverse populations and utilize longitudinal designs to explore the long‐term effects of caregiver support on recovery. Additionally, future studies could examine the impact of NMM workshops interventions on aspects of family functioning and caregivers' outcomes, specifically identifying which improved dimensions mediate the overall observed improvements.

In conclusion, the present study highlights the potential relevance of family support in the treatment of adult individuals with AN, suggesting that the integration of psychoeducational caregiver interventions, such as NMM training workshops, may be beneficial within adult ED services. When combined with CBT‐E, such interventions may not only enhance treatment outcomes but also help address relational and emotional barriers that can limit the effectiveness of individual therapy. Including structured family support in treatment plans could help address a gap in the current literature and may contribute to a more comprehensive, integrative treatment model that acknowledges the possible role of caregivers in the recovery process.

## Author Contributions

**Cristiano Dani:** conceptualization, methodology, data curation, formal analysis, writing – review and editing, writing – original draft. **Eleonora Rossi:** conceptualization, methodology, validation, writing – review and editing. **Emanuele Cassioli:** conceptualization, methodology, supervision, validation, writing – review and editing. **Valentina Zofia Cordasco:** data curation, writing – review and editing. **Alice Roscioli:** data curation, writing – review and editing. **Giulia Selvi:** data curation, writing – review and editing. **Livio Tarchi:** methodology, writing – review and editing. **Luca Zompa:** methodology, writing – review and editing. **Sandra Moretti:** supervision, writing – review and editing. **Maria Rita Troiani:** validation, writing – review and editing. **Stefano Lucarelli:** visualization, writing – review and editing. **Valentina Cardi:** visualization, writing – review and editing. **Valdo Ricca:** validation, visualization, writing – review and editing. **Giovanni Castellini:** conceptualization, supervision, validation, visualization, writing – review and editing.

## Conflicts of Interest

The authors declare no conflicts of interest.

## Supporting information


**Figure S1:** CONSORT flow diagram of recruitment process.
**Figure S2:** Contour plots illustrating the interaction between‐group membership (NMM vs. no NMM), time (days), and clinical outcomes (BMI, EDE‐Q global score, BUT‐A GSI, DERS total score, SCL‐90‐R GSI, TAS‐20 total score).
**Figure S3:** 3D surface plots illustrating the interaction between‐group membership (NMM vs. no NMM), time (days), and clinical outcomes (BMI, EDE‐Q global score, BUT‐A GSI, DERS total score, SCL‐90‐R GSI, TAS‐20 total score).
**Table S1:** Secondary analysis: duration of illness.
**Table S2:** Secondary analysis: SSRI medication use.
**Table S3:** Secondary analysis: number of CBT‐E sessions.

## Data Availability

The data that support the findings of this study are available from the corresponding author upon reasonable request.
