# Supplementary material for: A Non‐Randomized Comparison of the Addition of New Maudsley Model Workshops for Parents of Adult Women Receiving Multidisciplinary Care for Anorexia Nervosa
Source: Int J Eat Disord. 2025 Oct 15;59(1):179–89. doi: 10.1002/eat.24577 (PMC12773670; doi:10.1002/eat.24577)
Supplement: Supplementary file 1 — Figure S1: CONSORT flow diagram of recruitment process. Figure S2: Contour plots illustrating the interaction between‐group membership (NMM vs. no NMM), time (days), and clinical outcomes (BMI, EDE‐Q global score, BUT‐A GSI, DERS total score, SCL‐90‐R GSI, TAS‐20 total score). Figure S3: 3D surface plots illustrating the interaction between‐group membership (NMM vs. no NMM), time (days), and clinical outcomes (BMI, EDE‐Q global score, BUT‐A GSI, DERS total score, SCL‐90‐R GSI, TAS‐20 total score). Table S1: Secondary analysis: duration of illness. Table S2: Secondary analysis: SSRI medication use. Table S3: Secondary analysis: number of CBT‐E sessions. [file EAT-59-179-s001.docx]

**Supplementary Materials**

**Figure S1. CONSORT Flow Diagram of recruitment process**

**
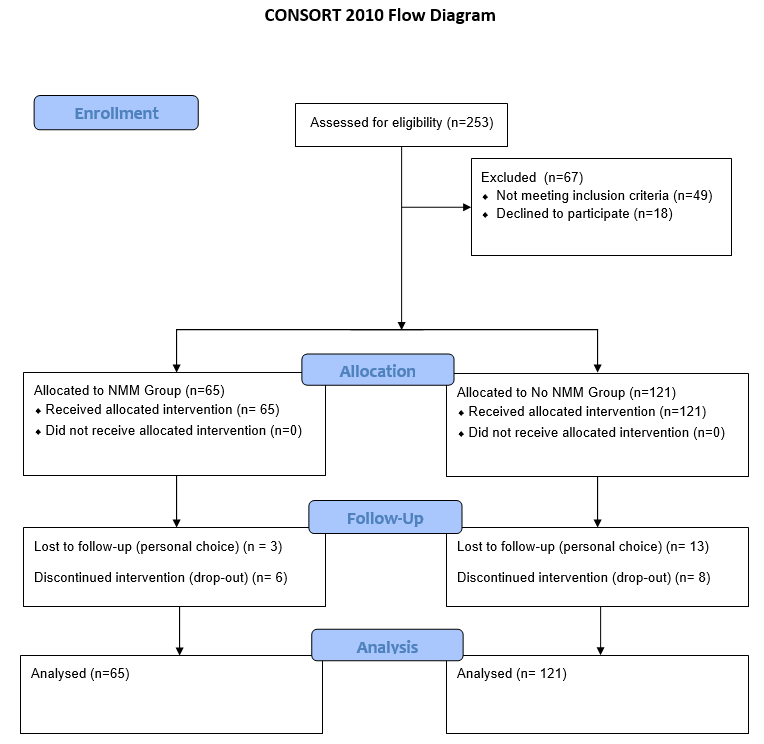
**

**Figure S2. Contour plots illustrating the interaction between group membership (NMM vs No NMM), time (Days), and clinical outcomes (BMI, EDE-Q Global Score, BUT-A GSI, DERS Total Score, SCL-90-R GSI, TAS-20 Total Score).**

**
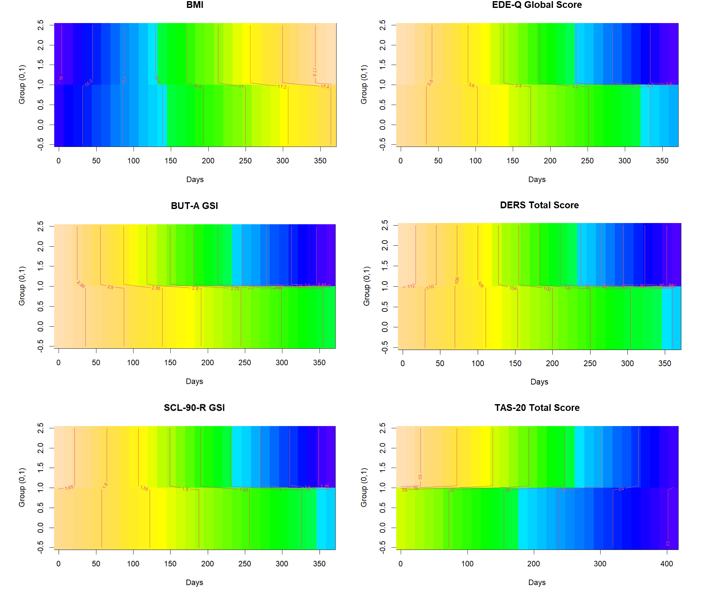
**

*Legend:* Contour plots represent the estimated values over time, with blue indicating lower levels and yellow representing higher levels. Group 1 represents the NMM Group, and Group 0 represents the No NMM group.

*Abbreviations:* BMI: body mass index; EDE-Q: Eating Disorder Examination Questionnaire; BUT-A GSI: Body Uneasiness Test-A Global Severity Index; DERS: Difficulties in Emotion Regulation Scale; SCL-90-R: Symptom Checklist-90-Revised, GSI: Global Severity Index; TAS-20: Toronto Alexithymia Scale - 20.

**Figure S3. 3D surface plots illustrating the interaction between group membership (NMM vs No NMM), time (Days), and clinical outcomes (BMI, EDE-Q Global Score, BUT-A GSI, DERS Total Score, SCL-90-R GSI, TAS-20 Total Score).**


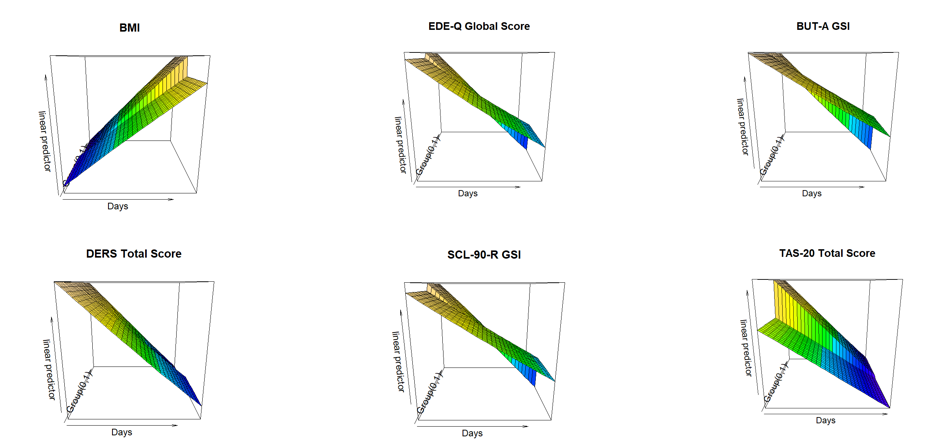


*Legend:* Perspective plots provide a detailed visualization of the modeled trajectories, highlighting the moderating effect of group membership on outcome progression. Group 1 represents the NMM Group, and Group 0 represents the No NMM group.

*Abbreviations:* BMI: body mass index; EDE-Q: Eating Disorder Examination Questionnaire; BUT-A GSI: Body Uneasiness Test-A Global Severity Index; DERS: Difficulties in Emotion Regulation Scale; SCL-90-R: Symptom Checklist-90-Revised, GSI: Global Severity Index; TAS-20: Toronto Alexithymia Scale - 20.

**Table S1. Secondary analysis: duration of illness**

|  | **Group fixed effect** |
| --- | --- |
| BMI (kg/m²) | -0.03, [-0.08, 0.01] |
| EDE-Q Global Score | 0.04, [-0.02, 0.09] |
| BUT-A GSI | 0.00, [-0.03, 0.04] |
| DERS Total Score | 0.30, [-0.89, 1.50] |
| SCL-90-R GSI | 0.00, [-0.02, 0.03] |
| TAS-20 Total Score | -0.19, [-0.62, 0.24] |

*Notes:* Values are GAMM estimates for the fixed effect of group (NMM vs. No NMM) on each outcome, reported as β with 95% confidence intervals, adjusted for baseline values, age, and BMI.

*Abbreviations*: BMI: body mass index; EDE-Q: Eating Disorder Examination Questionnaire; BUT-A GSI: Body Uneasiness Test-A Global Severity Index; DERS: Difficulties in Emotion Regulation Scale; SCL-90-R: Symptom Checklist-90-Revised, GSI: Global Severity Index; TAS-20: Toronto Alexithymia Scale - 20.

**Table S2. Secondary analysis: SSRI medication use**

|  | **Group fixed effect** |
| --- | --- |
| BMI (kg/m²) | 0.01,[-0.23, 0.26] |
| EDE-Q Global Score | 0.01, [-0.06, 0.01] |
| BUT-A GSI | 0.00, [-0.14, 0.15] |
| DERS Total Score | -0.81, [-0.72, 0.90] |
| SCL-90-R GSI | -0.01, [-0.11, 0.09] |
| TAS-20 Total Score | -0.05, [-0.58, 0.49] |

*Notes:* Values are GAMM estimates for the fixed effect of group (NMM vs. No NMM) on each outcome, reported as β with 95% confidence intervals, adjusted for baseline values, age, and BMI.

*Abbreviations*: BMI: body mass index; EDE-Q: Eating Disorder Examination Questionnaire; BUT-A GSI: Body Uneasiness Test-A Global Severity Index; DERS: Difficulties in Emotion Regulation Scale; SCL-90-R: Symptom Checklist-90-Revised, GSI: Global Severity Index; TAS-20: Toronto Alexithymia Scale - 20.

|  | **Group fixed effect** |
| --- | --- |
| BMI (kg/m²) | -0.01, [-0.03, 0.03] |
| EDE-Q Global Score | -0.00, [-0.04, 0.02] |
| BUT-A GSI | -0.02, [-0.03, 0.00] |
| DERS Total Score | 0.19, [-0.82, 0.60] |
| SCL-90-R GSI | -0.00, [-0.02, 0.01] |
| TAS-20 Total Score | -0.06, [0.50, 0.39] |

**Table S3. Secondary analysis: number of CBT-E sessions**

*Notes:* Values are GAMM estimates for the fixed effect of group (NMM vs. No NMM) on each outcome, reported as β with 95% confidence intervals, adjusted for baseline values, age, and BMI.

*Abbreviations*: BMI: body mass index; EDE-Q: Eating Disorder Examination Questionnaire; BUT-A GSI: Body Uneasiness Test-A Global Severity Index; DERS: Difficulties in Emotion Regulation Scale; SCL-90-R: Symptom Checklist-90-Revised, GSI: Global Severity Index; TAS-20: Toronto Alexithymia Scale - 20.
